# Defective Lung Macrophage Function in Lung Cancer±Chronic Obstructive Pulmonary Disease (COPD/Emphysema)-Mediated by Cancer Cell Production of PGE2?

**DOI:** 10.1371/journal.pone.0061573

**Published:** 2013-04-26

**Authors:** Francis C. Dehle, Violet R. Mukaro, Craig Jurisevic, David Moffat, Jessica Ahern, Greg Hodge, Hubertus Jersmann, Paul N. Reynolds, Sandra Hodge

**Affiliations:** 1 Lung Research Laboratory, Hanson Institute, Adelaide, South Australia, Australia; 2 Department of Thoracic Medicine, Royal Adelaide Hospital, Adelaide, South Australia, Australia; 3 Department of Cardiothoracic Surgery, Royal Adelaide Hospital, Adelaide, South Australia, Australia; 4 Department of Surgical Pathology, SA Pathology, Adelaide, South Australia, Australia; 5 Department of Medicine, University of Adelaide, Adelaide, South Australia, Australia; University of Oslo, Norway

## Abstract

In chronic obstructive pulmonary disease (COPD/emphysema) we have shown a reduced ability of lung and alveolar (AM) macrophages to phagocytose apoptotic cells (defective ‘efferocytosis’), associated with evidence of secondary cellular necrosis and a resultant inflammatory response in the airway. It is unknown whether this defect is present in cancer (no COPD) and if so, whether this results from soluble mediators produced by cancer cells.

We investigated efferocytosis in AM (26 controls, 15 healthy smokers, 37 COPD, 20 COPD+ non small cell lung cancer (NSCLC) and 8 patients with NSCLC without COPD) and tumor and tumor-free lung tissue macrophages (21 NSCLC with/13 without COPD). To investigate the effects of soluble mediators produced by lung cancer cells we then treated AM or U937 macrophages with cancer cell line supernatant and assessed their efferocytosis ability. We qualitatively identified Arachidonic Acid (AA) metabolites in cancer cells by LC-ESI-MSMS, and assessed the effects of COX inhibition (using indomethacin) on efferocytosis.

Decreased efferocytosis was noted in all cancer/COPD groups in all compartments. Conditioned media from cancer cell cultures decreased the efferocytosis ability of both AM and U937 macrophages with the most pronounced effects occurring with supernatant from SCLC (an aggressive lung cancer type). AA metabolites identified in cancer cells included PGE2. The inhibitory effect of PGE2 on efferocytosis, and the involvement of the COX-2 pathway were shown.

Efferocytosis is decreased in COPD/emphysema and lung cancer; the latter at least partially a result of inhibition by soluble mediators produced by cancer cells that include PGE2.

## Introduction

COPD is predicted to become the third most prevalent cause of death worldwide by 2020 [1]. The carcinogenic effects of tobacco smoke have been well-described; however, smokers with COPD have a higher risk of developing lung cancer than do smokers without COPD [2] even when corrected for tobacco consumption. The prevalence of COPD and lung cancer is projected to increase in the coming decades due to continued exposure to risk factors and an ageing population. These statistics are alarming as lung cancer is responsible for more cancer-related deaths than colon, breast and prostate cancers combined [3]. Although smoking cessation remains paramount, many new lung cancers are detected in ex- rather than current smokers. There is an urgent need to better understand the links between smoking, COPD and lung cancer to enable novel therapeutic or preventative strategies.

We have shown that alveolar macrophages (AM) in COPD are defective in their capacity to phagocytose apoptotic cells (efferocytosis) [4]–[7], which has the potential to contribute to the excess apoptotic material that we have reported in the COPD airway [8]. This un-cleared material can then undergo secondary necrosis and perpetuate the inflammatory response [8]. At this stage it is unknown whether there is also a defect in the efferocytosis ability of *lung tissue* macrophages from COPD subjects, although our findings with a smoking mouse model suggest that this may be the case [9], [10]. Also unknown is whether there are defects in the efferocytosis ability of AM and lung tissue/tumor-associated macrophages from patients with cancer in the absence of COPD, and if so, whether soluble mediators produced by the lung cancer cells inhibit efferocytosis.

Evidence suggests that un-cleared apoptotic material that results from defective efferocytosis, in addition to its inflammatory effects, can stimulate the influx of regulatory T lymphocytes which exert immuno-suppressive effects against anti-tumor immunity and thus contribute to the tumor's ability to escape eradication [11]. Whether defects in the mechanisms that lead to defective efferocytosis in COPD also play a direct role in creating a pro-tumour environment and contribute to an increased susceptibility to lung cancer is unknown.

Cancer cells are directly immunosuppressive by releasing pro-inflammatory mediators including Arachidonic Acid (AA) metabolites such as prostaglandins (PGE) [12]. PGE is released in cells expressing constitutive cyclooxygenase-2 (COX-2), regulated by liberation of AA by phospholipase A 2 followed by metabolism by COX. PGE2 has been shown to decrease phagocytosis of bacteria via an E-prostanoid 2 receptor -mediated process [13].

We therefore investigated efferocytosis in AM from controls, smokers, current/ex-smoker COPD subjects and patients with non small cell lung cancer (NSCLC) with/without COPD. We also investigated macrophages from tumor and tumor-free tissues obtained at lobectomy from patients with NSCLC with/without COPD.

We then investigated whether soluble mediators produced by lung cancer cells would directly suppress macrophage efferocytic ability. We assessed the effect of supernatant from cancer cell cultures on efferocytosis then applied electrospray ionization tandem mass spectrometry (LC-ESI-MSMS) analysis for AA metabolites in lung cancer cells. We finally investigated the potential for the COX inhibitor indomethacin to improve efferocytosis.

## Materials and Methods

### Subject population and bronchoalveolar lavage samples (BAL)

Cancer and COPD groups included patients undergoing bronchoscopy at the Royal Adelaide Hospital (RAH) to investigate suspected lung cancer. We included a small group of patients with lung cancer without COPD to further dissect the effects of lung cancer alone versus COPD. Controls, smokers and some COPD subjects were recruited from our volunteer database. Controls had normal lung function and no history of lung disease, cancer or allergy. We have previously found no significant differences in efferocytosis between never-smoker and ex-smoker controls and therefore grouped them together as ‘control group’. Written informed consent was obtained with ethics approval granted by the RAH.

Flexible bronchoscopy was performed and bronchoalveolar lavage samples (BAL) obtained according to recommendations by the American Thoracic Society as previously reported [4]–[7], [14]. For patients with cancer, the BAL was taken well away from the affected region of the lung. Spirometric assessment of predicted values and lower limits of normality (LLN) for forced vital capacity (FVC), forced expiratory volume in first second (FEV(1)), and FEV(1)/FVC ratio was performed and the diagnosis of COPD established using the Global Initiative for Chronic Obstructive Lung Disease (GOLD) criteria (FEV1/FVC <70%) with x-ray and clinical correlation [15]. For three patients where FEV1/FVC was slightly higher that LLN, the presence of emphysema was confirmed with high resolution CT or x-ray; these patients were therefore included. Cancer patients all had NSCLC diagnosed on the basis of World Health Organization criteria [16] and were further categorized on the presence of COPD. Microbiological colonization and differential cell counts were assessed by SA Pathology (Adelaide, South Australia).

We investigated efferocytosis in AM from 24 controls, 15 healthy smokers, 20 current/16 ex-smoker COPD subjects and 8 patients with NSCLC without COPD and 17 with COPD (Table 1). We also investigated macrophages from tumor and tumor-free tissues obtained at lobectomy from patients with NSCLC (21 with/13 without COPD) (Table 2).

**Table 1:** BAL patient demographics.

Subjects	Age (range)	Pack Yrs	Pre BD		Post BD		LLN	Smokercur/ex/n	N M/F	VolmL	WCC10^9^/L	Mac%	Ly(%)
			FEV_1_ (% pred)	FEV_1_/FVC	FEV_1_ (% pred)	FEV_1_/FVC	FEV_1_/FVC						
Controls	57 [44–75]	7[3]	101.6 [2.9]	79.5 [1.9]	103.2 [4.0]	79.5 [2.0]	68.1 [0.6]	0/8/16	10/14	67.7 [3.8]	0.21 [.04]	77.4 [4.0]	11.6 [1.6]
Healthy smokers	50 [29–66]	34[7]	92.3 [2.6]	74.8 [1.4]	99.6 [2.6]	77.7 [2.2]	69.4 [0.9]	13/0/0	9/6	66.3 [4.6]	1.13 [.55]*	85.8 [3.2]	9.5 [2.5]
COPD Cur-smoker	60 [37–75]	49[7]*	71.8 [3.0]*	60.0 [2.0]*	77.6 [3.3]	60.9 [2.0]	67.2 [0.6]	20/0/0	11/9	49.9 [3.5]*	0.36 [.10]	81.6 [4.2]	6.6 [1.8]
COPD ex-smoker	64 [55–73]*	58[15]*	71.9 [4.1]*	58.4 [3.3]*	76.8 [5.0]	58.3 [2.4]	66.6 [1.0]	0/16/0	10/6	51.3 [6.3]	0.35 [.05]	74.4 [8.9]	8.6 [1.7]
COPD +cancer	67 [55–77]*	35[7]	66.5 [2.0]*	62.3 [2.3]*	69.2 [2.3]	63.3 [2.5]	65.3 [0.4]	11/9/0	14/6	55.4 [5.2]	0.20 [.07]	86.3 [3.5]	5.9 [1.2]
Cancer no COPD	61 [44–75]	11[7]	91.8 [5.3]	78.6 [0.9]	94.1 [6.7]	89.7 [6.9]	66.8 [1.0]	4/1/3	4/4	49.2 [9.8]*	0.34 [.07]	73.2 [11.4]	6.6 [1.4]

**Table 2:** Tissue/tumor patient demographics.

Subjects	Age yrs	Pack yrs	Smoker Cur/ex/n	Pre BD		Post BD		LLN	N M/F	Cancer type				
				FEV_1_% pred	FEV_1_/FVC	FEV_1_% pred	FEV_1_% pred	FEV_1_/FVC		Adeno	Squam	Adenosquam	Undiff	Largecell
Cancer+ COPD	69 [2]	42.5 [6]	10/10/0	66.6 [2.1]	67.7 [2.2]	59.7 [3.8]	62.8 [2.8]	64.52 [0.54]	14/7	7	10	1	1	2
Cancer no COPD	66 [2.5]	17 [5]	5/5/3	90.3 [3.6]	90.0 [3.4]	91.0 [3.5]	80.0 [2.0]	65.2 [0.6]	8/5	8	4	0	0	1

Values shown as mean [SEM]. COPD, chronic obstructive pulmonary disease; yrs, years; FEV_1_, forced expiratory volume in 1 min; FVC, forced vital capacity; BD, bronchodilator; LLN, lower limit of normality; Cur, current-smoker; ex, ex-smoker; n, never smoker; adeno, adenocarcinoma; squam, squamous; * significantly (p<0.05) different compared to control.

### Lung tissues samples

Tumor and non-tumor lung tissue were obtained from patients undergoing lobectomy at the Department of Cardiothoracic Surgery, RAH, following informed consent, as previously described [17], [18]. Tumor was obtained using a biopsy needle, while biopsies were collected from non-tumor (‘normal’) areas well away from the cancer (approximately 5 mm×5 mm in size). Biopsies were performed by a qualified surgical pathologist, ensuring that representative tumor and normal tissue samples were collected. A ‘Medimachine’ tissue disaggregator (BD) was applied to prepare single cell suspensions from lung tissue as previously described [9], [17].

Samples were categorized as ‘Control (non-tumor)’ (non-cancer area from patients with cancer/no COPD), ‘Control (tumor)’ (cancer site from patients with cancer/no COPD), ‘COPD (non-tumor)’ (non-cancer area from patients with cancer+COPD) and ‘COPD (tumor)’ (cancer site from patients with cancer+COPD).

### Preparation of macrophages

Macrophages from BAL and tissue were isolated as reported [4]–[7] (detailed in Supporting Information S1).

### Immunological reagents and cell lines

Immunological reagents, cell lines (normal human bronchial epithelial cells (16HBE), lung adenocarcinoma (H2009, H1466), and small-cell carcinoma (SBC-1)) and culture/stimulation conditions are detailed in Supporting Information S1.

### Efferocytosis ability of BAL, tissue and U937 macrophages

Efferocytosis was investigated by flow cytometry as reported [4]–[7] and detailed in Supporting Information S1.

### LC-ESI-MSMS analysis for AA metabolites in cultured lung cancer cells

20 µL 4×10^5^ cancer cells or primary airway epithelial cells obtained by bronchial brushings at bronchoscopy (as control) were assessed for the presence of AA and COX derived AA metabolites (PGE2, PGD2, PGF2α, TXN2, 6ketoPGF1, 11-hydroxyeicosatetraenoic acid (11-HETE), thromboxane B2 (TxB2); lipoxygenase (LOX) products (12- and 5- HETE); eicosapentaenoic acid (EPA), docosahexaenoic acid (DHA), (8-HODE, LTB4), 12(5)TETE, PGB2 and 13(5)HODE using LC-ESI-MSMS as detailed in Supporting Information S1.

### Effect of prostaglandins on efferocytosis

Preparation of conditioned media from cancer cell lines is described in Supporting Information S1. We firstly investigated the effects of endogenous PGE2 on efferocytosis. U937 macrophages were treated with varying concentrations of PGE2 (0.1–10 µM) prior to assessment of efferocytosis. Having shown a dose-dependent effect on efferocytosis we then assessed the potential effects of PGE2 produced by lung cancer cells on efferocytosis. Cells were treated with supernatant from SBC-1 cells±the COX inhibitor indomethacin (1–10 µM) before assessing the effects of cancer cell supernatant on efferocytosis.

### Statistical analysis

Kruskal-Wallis and Spearman's rank correlation tests were applied. For in vitro experiments, ‘treatment’ results were compared with controls [RPMI media treatment] using one-way ANOVA and Dunnett's test.

## Results

### Patient demographics

BAL volumes were significantly reduced for current-smoker COPD and cancer (no COPD) groups vs. controls (Table 1). Total WCC was increased in current-smokers without COPD vs. controls (Table 1). Typical BAL yielded 75– 95% AM. There were no significant differences in% AM or lymphocytes in any patient group vs. controls (Table 1) although there was a non-significant trend for a reduced% of lymphocytes in the cancer groups.

### BAL and tissue macrophage efferocytosis

**Figure 1:**
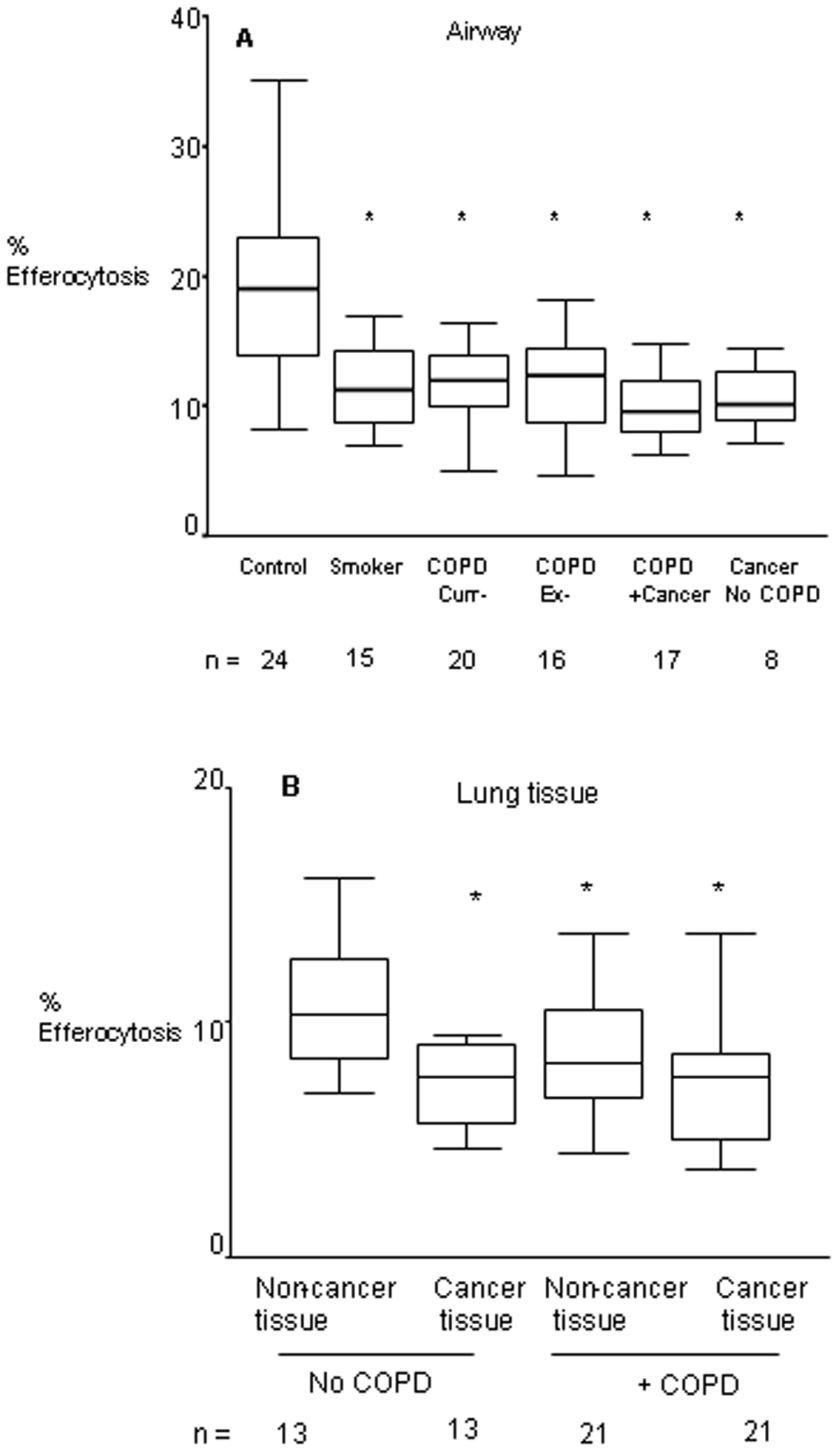
Efferocytosis ability of alveolar and lung tissue macrophages. **A.** Efferocytosis of BAL-derived alveolar macrophages was assessed for controls (‘C’), smokers, current- and ex- smokers with COPD (‘COPD Cur’ and ‘COPD Ex’), COPD subjects with lung cancer (‘COPD Cancer’) and patients with lung cancer and no COPD (‘Cancer’); **B.** Tissue from Controls (‘C Non-Tumor’) (non-cancer area from patients with cancer/no COPD), ‘COPD Non-Tumor’ (non-cancer area from patients with cancer+COPD), ‘COPD Tumor’ (cancer site from patients with cancer+COPD) and ‘Control Tumor’ (cancer site from patients with cancer/no COPD). *significantly (p<0.05) lower expression vs. controls (non-parametric Kruskal-Wallis test)Box plots present median±25th and 75th percentiles (solid box) with the 10th and 90th percentiles shown by whiskers outside the box.

Significantly decreased efferocytosis was noted in cancer and COPD groups for both AM and lung tissue macrophages, and for AM from healthy smokers. Efferocytosis varied between compartments, with AM showing a greater phagocytic ability than tissue or tumour macrophages, regardless of COPD status (COPD: (mean±SEM) BAL 12.83±0.87 >non-tumour tissue 8.67%±0.52 >7.48%±0.78; Non-COPD: BAL 20.30±1.79 >non-tumour tissue 9.45%±0.48 >7.80%±0.68). In COPD, efferocytosis was decreased independently of smoking status and lung cancer (Figure 1). For tissue macrophages, efferocytosis was decreased in COPD to the same extent in tissue adjacent to tumour and in unaffected areas, while in cancer patients without COPD, efferocytosis was decreased only in tumour areas. There were no significant differences in efferocytosis between BAL macrophages from COPD vs. COPD patients with cancer (Figure 1) or between COPD non-cancer vs. COPD cancer tissue macrophages

### Lung cancer cell line supernatant inhibit efferocytosis

**Figure 2:**
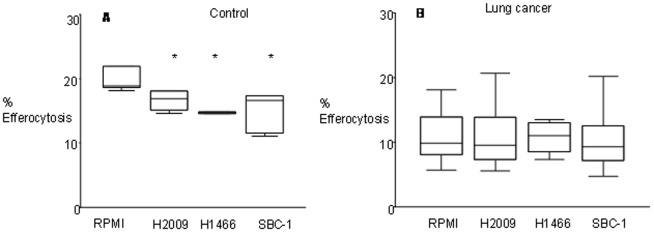
Effect of cancer cell line supernatants on efferocytosis. **A.** Effect of cancer cell line supernatants on the phagocytosis of apoptotic bronchial epithelial cells by alveolar macrophages. Macrophages from (A) control subjects or (B) subjects with lung cancer were incubated in normal RPMI media or cancer cell line supernatants (H2009, H1466, SBC1) for 24 hrs prior to phagocytosis assay. Values are presented as percentage of macrophages ingesting apoptotic cells *, p< 0.05 compared with RPMI media treatment (n = 5 experiments performed in triplicate; one-way ANOVA, Dunnett’s test). Data presented as box plots as described in Figure 1.

AM from controls or subjects with lung cancer were co-cultured with supernatant from NSCLC (H2009, H1466), and SCLC (SBC-1) cells. For AM from controls, lung cancer supernatant significantly decreased efferocytosis by 29%–33% (Figure 2). Interestingly, supernatant did not affect the (already significantly reduced) efferocytosis ability of AM from lung cancer subjects.

### Lung cancer cell lines contain AA metabolites

All three lung cancer cell lines (A549, H2009, SBC-1) showed detectable levels of PGE2, PGD2, PGF2α, TXN2 and 6ketoPGF1. 11-HETE, TxB2; 12- and 5- HETE; EPA, DHA), 8-HODE, LTB4, 12(5)TETE, PGB2 and 13(5)HODE were not identified. Based on the presence of PGE2 we then investigated the effects of the COX inhibitor, indomethacin, on efferocytosis.

### PGE-2 inhibits efferocytosis

**Figure 3:**
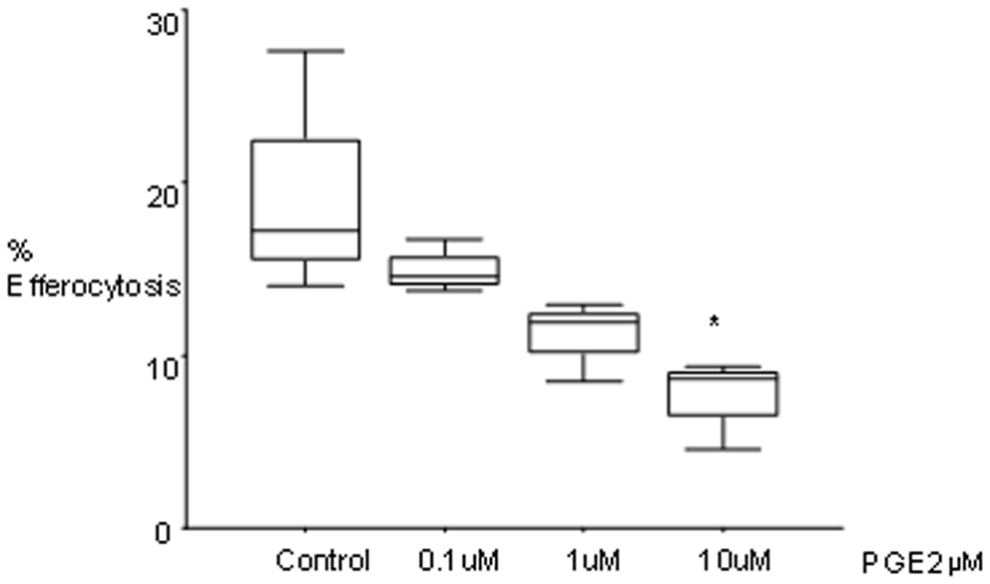
Effect of prostaglandins on efferocytosis. Prostaglandins inhibit efferocytosis and at least partially mediate the inhibitory effect of cancer cell line supernatants on the phagocytosis of apoptotic bronchial epithelial cells by U937 cells. U937 cells were incubated with varying concentrations of PGE2 for 18 hours and efferocytosis assessed (n = 3 triplicate experiments; one-way ANOVA, Dunnett’s test).

**Figure 4:**
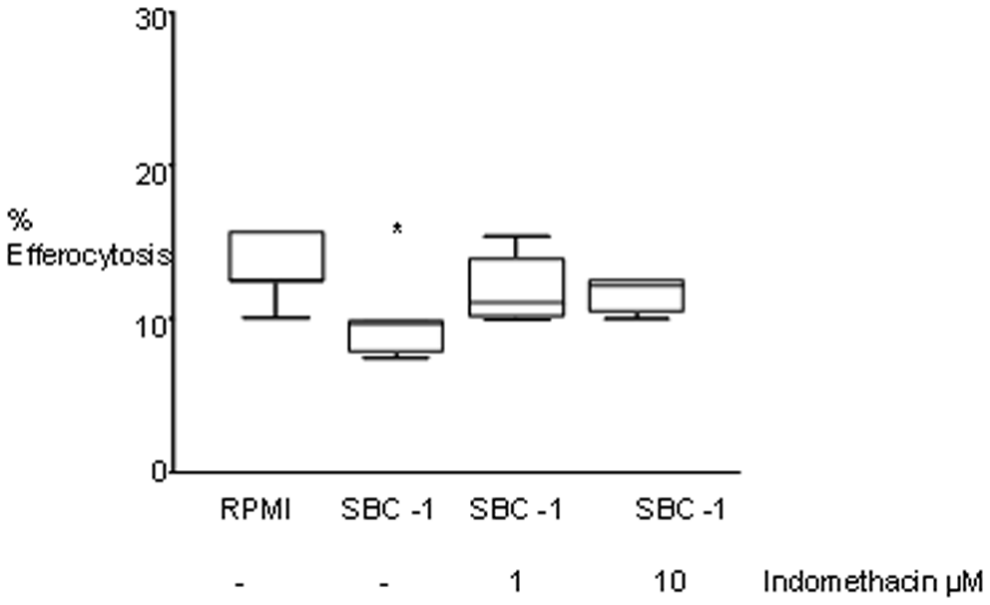
Prostaglandin inhibition of efferocytosis involves COX-2. U937 cells were incubated in normal RPMI media or SBC-1 supernatant that had been treated with indomethacin. Following 24 hrs incubation, an efferocytosis assay was performed. (n = 5 experiments performed in triplicate). Data presented as box plots as described in Figure 1. Values are presented as percentage of macrophages ingesting apoptotic cells *, p<0.05 compared with RPMI media treatment (one-way ANOVA, Dunnett’s test).

Exposure of U937 cells to PGE2 dose-dependently reduced their efferocytosis ability in a dose-dependent manner, with maximum and significant inhibition occurring in the presence of 10 µM PGE2 (Figure 3). To investigate the potential effects of PGE2 produced by lung cancer cells (via the COX-2/PGE-2 pathway) on efferocytosis, we then treated SBC1 cells with the COX inhibitor indomethacin. SBC1 cells were selected for these experiments as they had the greatest effect on efferocytosis. Indomethacin had no negative effects on cell growth or viability of the SBC-1 cells at the concentrations used (data not shown). We determined the effect of SBC-1 cell supernatant on PMA-differentiated U937 macrophages. Supernatant from SBC-1 cells grown in the absence of indomethacin significantly decreased the efferocytosis ability of U937 cells by 36% (Figure 4). This decrease was partially inhibited by indomethacin (1 µM: 21.3% increase vs. cancer cell supernatant; 10 µM: 25.74% increase).

## Discussion

A better understanding of the links between COPD and lung cancer is critical to advise novel therapeutic or preventative strategies. We report that the efferocytosis ability of both alveolar and lung tissue macrophages is deficient in patients with lung cancer with or without COPD (in COPD, independently of smoking status and lung cancer). In COPD, efferocytosis was decreased to a similar extent in tissue macrophages adjacent to the tumour and in unaffected areas, while in cancer patients without COPD, the defect was noted only in the tumour-associated macrophages, implying that COPD and cancer both result in impaired efferocytosis, by partly independent mechanisms. Un-cleared apoptotic material is likely to be important in the progression of lung cancer as this material has been shown to have pro-inflammatory effects [19] and contribute to a tumor’s ability to escape eradication by several mechanisms including stimulating of an influx of regulatory T lymphocytes [12]. Patients treated with rituximab, a drug which acts in part by opsonizing cells and increasing phagocytosis had a positive prognostic value despite the high number of tumor-associated macrophages [20], [21].

Phagocytosis may be suppressed via release of soluble mediators including PGE-2 by lung cancer cells. In mesothelioma; cancer cells co-cultured with macrophages induced a decrease in phagocytosis but an increase in the PGE-2 release [22]. In the present study we therefore applied LC-ESI-MSMS to determine whether lung cancer cells produced various prostaglandins that could directly inhibit efferocytosis. All three lung cancer cell lines produced detectable levels of AA metabolites including PGE-2, consistent with another study [23] of NSCLC cell lines A549 and H1299. Inhibition of COX with indomethacin at least partially negated the suppressive effects on efferocytosis, implicating an effect of the COX-2/PGE-2 pathway although there are clearly other additional factors involved.

Interestingly, the most significant suppressive effects were found using the SCLC cell line, SBC-1 and we can hypothesize that this may be representative of the more aggressive nature of this lung cancer type. A previous study of COX-2 expression in primary NSCLC demonstrated the highest levels of both mRNA and protein in adenocarcinoma cells compared with large cell and squamous cell carcinoma [24]. They did not, however, investigate SCLC. It will be of interest to determine whether AA metabolites inhibit the efferocytosis function of airway and tumor-associated macrophages to a similar extent, as we found that lung cancer cell supernatant had a significant suppressive effect on the efferocytic ability of macrophages from control subjects but did not affect the (already significantly reduced) efferocytic ability of macrophages from subjects with lung cancer.

Drawbacks of the study are that we were not able to compare BAL and lung tissue compartments for individual patients; we are however assessing this in our murine emphysema model in ongoing studies. There are also obvious ethical drawbacks of obtaining fresh ‘normal’ lung. Although we collected our ‘control’ lung tissue by biopsy taken well away from the area of cancer in patients without COPD, the possibility still remains that the presence of cancer may have influenced some of our findings and murine cancer models are required to clarify this. The number of NSCLC patients without COPD was relatively low (BAL from 8 patients with NSCLC and paired cancer and cancer-free tissue from 13 patients was obtained over a 2 year period) which precludes firm conclusions based on analysis of data from this group of patients. Of the about 200 patients with NSCLC, which our unit sees annually only a small percentage (∼15%) are considered for surgery (Stages I and II), and of those many are medically inoperable. In our operable patients with NSCLC the proportion of patients without COPD is very low, about 10%.

In conclusion we have shown decreased efferocytosis in airway and lung tissue in both COPD and lung cancer, and the inhibition of efferocytosis via release of soluble prostaglandins by lung cancer cells. These defects may be a potential immune evasion mechanism in lung cancer.

## Supporting Information

Supporting Information S1 (DOC)

## References

[1] Access Economics Report for the Australian Lung Foundation (2008) Economic Impact of COPD and cost effective solutions. Available: http://www.lungfoundation.com.au/lung-information/publications/economic-impact-of-copd-2008. Accessed 2012 Jan 28.

[2] Skillrud DM (1986) COPD: causes, treatment, and risk for lung cancer. Compr Ther 12(11): 13–16. 2878761

[3] Fong KM, Sekido Y, Gazdar AF, Minna JD (2003) Lung cancer. 9: Molecular biology of lung cancer: clinical implications. Thorax 58(10): 892–900. 14514947 10.1136/thorax.58.10.892 PMC1746489

[4] Hodge S, Hodge G, Scicchitano R, Reynolds PN, Holmes M (2003) Alveolar macrophages from subjects with chronic obstructive pulmonary disease are deficient in their ability to phagocytose apoptotic airway epithelial cells. Immunol Cell Biol 81(4): 289–296. 12848850 10.1046/j.1440-1711.2003.t01-1-01170.x

[5] Hodge S, Hodge G, Brozyna S, Jersmann H, Holmes M, et al (2006) Azithromycin increases phagocytosis of apoptotic bronchial epithelial cells by alveolar macrophages. Eur Respir J 28(3): 486–495. 16737992 10.1183/09031936.06.00001506

[6] Hodge S, Hodge G, Ahern J, Jersmann H, Holmes M, et al (2007) Smoking alters alveolar macrophage recognition and phagocytic ability: implications in chronic obstructive pulmonary disease. Am J Respir Cell Mol Biol 37(6): 748–755. 17630319 10.1165/rcmb.2007-0025OC

[7] Hodge S, Hodge G, Jersmann H, Matthews G, Ahern J, et al (2008) Azithromycin improves macrophage phagocytic function and expression of mannose receptor in chronic obstructive pulmonary disease. Am J Respir Crit Care Med 178(2): 139–148. 18420960 10.1164/rccm.200711-1666OC

[8] Hodge S, Hodge G, Holmes M, Reynolds PN (2005) Increased airway epithelial and T-cell apoptosis in COPD remains despite smoking cessation. Eur Respir J 25(3): 447–454. 15738287 10.1183/09031936.05.00077604

[9] Hodge S, Matthews G, Dean MM, Ahern J, Djukic M, et al (2009) Therapeutic Role for Mannose Binding Lectin in Cigarette Smoke-induced Lung Inflammation? Evidence from a Murine Model. Am J Respir Cell Mol Biol 42(2): 235–42. 19411612 10.1165/rcmb.2008-0486OC

[10] Hodge S, Matthews G, Mukaro V, Ahern J, Shivam A, et al (2011) Cigarette Smoke-induced Changes to Alveolar Macrophage Phenotype and Function is Improved by Treatment with Procysteine. Am J Respir Cell Mol Biol (44(5)) 673–81. 10.1165/rcmb.2009-0459OC 20595463

[11] Tuve S, Chen BM, Liu Y, Cheng TL, Toure P, et al (2007) Combination of tumor site-located CTL-associated antigen-4 blockade and systemic regulatory T-cell depletion induces tumor-destructive immune responses. Cancer Res 67(12): 5929–5939. 17575163 10.1158/0008-5472.CAN-06-4296

[12] Kim R, Emi M, Tanabe K (2006) Cancer immunosuppression and autoimmune disease: beyond immunosuppressive networks for tumour immunity. Immunology 119(2): 254–264. 17005005 10.1111/j.1365-2567.2006.02430.x PMC1782355

[13] Aronoff DM, Canetti C, Peters-Golden M (2004) Prostaglandin E2 inhibits alveolar macrophage phagocytosis through an E-prostanoid 2 receptor-mediated increase in intracellular cyclic AMP. J Immunol 173(1): 559–565. 15210817 10.4049/jimmunol.173.1.559

[14] Hodge SJ, Hodge GL, Holmes M, Reynolds PN (2004) Flow cytometric characterization of cell populations in bronchoalveolar lavage and bronchial brushings from patients with chronic obstructive pulmonary disease. Cytometry B Clin Cytom 61(1): 27–34. 15351979 10.1002/cyto.b.20020

[15] Global Initiative for Chronic Obstructive Lung Disease. Global strategy for the diagnosis, management, and prevention of chronic obstructive pulmonary disease. (2011) Available: www.goldcopd.org/. Accessed 2012 Jan 28.

[16] Travis WD, Brambilla E, Muller-Hermelink HK, Harris CC (2004) World Health Organization Classification of Tumours. Pathology and Genetics of Tumours of the Lung, Pleura, Thymus and Heart. Lyon: IARC Press.

[17] Soriano C, Mukaro V, Hodge G, Ahern J, Holmes M, et al (2012) Increased proteinase inhibitor-9 (PI-9) and reduced granzyme B in lung cancer: mechanism for immune evasion? Lung Cancer 77(1): 38–45. 22387007 10.1016/j.lungcan.2012.01.017

[18] Cordts F, Pitson S, Tabeling C, Gibbins I, Moffat DF, et al (2011) Expression profile of the sphingosine kinase signalling system in the lung of patients with chronic obstructive pulmonary disease. Life Sciences 89(21–22): 806–811. 21945191 10.1016/j.lfs.2011.08.018

[19] Vandivier RW, Henson PM, Douglas IS (2006) Burying the dead: the impact of failed apoptotic cell removal (efferocytosis) on chronic inflammatory lung disease. Chest 129(6): 1673–82. Review. 10.1378/chest.129.6.1673 16778289

[20] Leidi M, Gotti E, Bologna L, Miranda E, Rimoldi M, et al (2009) M2 macrophages phagocytose rituximab-opsonized leukemic targets more efficiently than m1 cells in vitro. J Immunol 182(7): 4415–4422. 19299742 10.4049/jimmunol.0713732

[21] Taskinen M, Karjalainen-Lindsberg ML, Nyman H, Eerola LM, Leppa S (2007) A high tumor-associated macrophage content predicts favorable outcome in follicular lymphoma patients treated with rituximab and cyclophosphamide-doxorubicin-vincristine-prednisone. Clin Cancer Res 13(19): 5784–5789. 17908969 10.1158/1078-0432.CCR-07-0778

[22] Izzi V, Chiurchiu V, D'Aquilio F, Palumbo C, Tresoldi I, et al (2009) Differential effects of malignant mesothelioma cells on THP-1 monocytes and macrophages. Int J Oncol 34(2): 543–550. 19148491

[23] Kempen EC, Yang P, Felix E, Madden T, Newman RA (2001) Simultaneous quantification of arachidonic acid metabolites in cultured tumor cells using high-performance liquid chromatography/electrospray ionization tandem mass spectrometry. Anal Biochem 297(2): 183–190. 11673886 10.1006/abio.2001.5325

[24] Watkins DN, Peroni DJ, Lenzo JC, Knight DA, Garlepp MJ, et al (1999) Expression and localization of COX-2 in human airways and cultured airway epithelial cells. Eur Respir J 13(5): 999–1007. 10414396 10.1034/j.1399-3003.1999.13e12.x

