# Supplementary material for: Defective Lung Macrophage Function in Lung Cancer±Chronic Obstructive Pulmonary Disease (COPD/Emphysema)-Mediated by Cancer Cell Production of PGE2?
Source: PLoS One. 2013 Apr 26;8(4):e61573. doi: 10.1371/journal.pone.0061573 (PMC3637201; doi:10.1371/journal.pone.0061573)
Supplement: Supporting Information S1 — (DOC) [file pone.0061573.s001.doc]

Supporting information files

**Defective efferocytosis in lung cancer ± COPD - mediated by cancer cell production of PGE2?**

Francis C. Dehle^1,2^, Violet R. Mukaro^1,2^, Craig Jurisevic^3^, David Moffat^4^, Jessica Ahern^1,2^, Greg Hodge^1,2,5^, Hubertus Jersmann^1,2,5^, Paul N. Reynolds^1,2,5^, Sandra Hodge^1,2,5^

**Preparation of macrophages**

Macrophages from BAL and dissagregated tissue were prepared as previously reported [4-7]. Briefly, macrophage numbers were adjusted to 4x10^5^/mL in RPMI supplemented with FCS, L-glutamine/penicillin/gentamicin (culture medium) (Gibco, Berlin, Germany) and macrophages purified by adhesion to plastic as previously reported (4-7). One mL aliquots were then adhered to 24 well culture plates for 2h, then fluid removed and the purified macrophages gently recovered using ice-cold culture medium and gentle pipetting.

**Immunological reagents**

For efferocytosis: CD33 [phycoerythrin cyanide-5 (PC-5)] (Coulter/Immunotech), mitotracker red [MTR] (Molecular Probes, Oregon, USA).

**Cell culture**

Immortalized normal human bronchial epithelial cells (16HBE), lung adenocarcinoma (H2009, H1466), and small-cell carcinoma (SBC-1) cell lines were grown in culture media. Cells were cultured in uncoated 75cm^2^ plastic flasks in an incubator with 95% air and 5% CO_2_ at 37^0^C. The medium was refreshed every 2-3 days. To obtain supernatant, the cells were cultured at a density of 5x10^5^ cells per/mL in 10 mL in a 75cm^2^ plastic flask. In some experiments the cells were treated with indomethacin (1 and 10µm). The cells were grown for 48h and the media was removed, filtered and immediately stored at -70^o^C.

U937 monocytic cells were maintained as described above. The cells were plated at a density of 4x10^5^ cells/ml and treated with PMA (50 ng/mL) for 48h. The media was removed and replaced with 500μL cell line supernatants or culture media (control). Cells were incubated for 24h and then analyzed for efferocytosis.

**Efferocytosis ability of BAL, tissue and U937 macrophages**

Efferocytosis was investigated as previously reported (4-7). Briefly, apoptotic airway epithelial cells (16HBE, at a density of 4x10^6^ cells/mL) were prepared by exposure to UV for 30 min, which induces apoptosis in >80% of cells (4), then stained with MTR (25 µg/ml) and 500uL aliquots added to adhered AM in 24-well plates. Following incubation for 90 min, the media was removed and ice-cold PBS added for 5 min. Macrophages were then removed using transfer pipettes, pelleted via centrifugation and stained with CD33 for 10 min. Autofluorescence and adherent but not phagocytosed 16HBE cells were quenched with crystal violet (4) and analyzed immediately using a FACSCalibur flow cytometer (BD).

**LC-ESI-MSMS analysis for AA metabolites in cultured lung cancer cells**

As a qualitative indication of the presence of AA metabolites in lung cancer cells that might be responsible for the inhibition of efferocytosis, we applied LC-ESI-MSMS analysis of cultured lung cancer cells. 20µL 4x10^5^ cancer cells or primary airway epithelial cells obtained by bronchial brushings at bronchoscopy (as control) were assessed for the presence of AA and COX derived AA metabolites (PGE2, PGD2, PGF2α, TXN2, 6ketoPGF1, 11-hydroxyeicosatetraenoic acid (11-HETE), thromboxane B2 (TxB2); lipoxygenase (LOX) products (12- and 5- HETE); eicosapentaenoic acid (EPA), docosahexaenoic acid (DHA), (8-HODE, LTB4), 12(5)TETE, PGB2 and 13(5)HODE using LC-ESI-MSMS. Cells were spotted onto 3mm diameter circles punched from S&S903 pre-treated filter paper in a 96 well, dried and stored at -20^o^C. LC-ESI-MSMS in negative ion was performed using an API 5000 instrument (AB SCIEX Foster City, Ca, USA) with a C8-column run in a gradient from 50 to 90% acetonitrile 0.1% formic acid. Filter paper spots were extracted with 50% acetonitile and formic acid (0.1%) for 20 min in a volume of 100µL containing the deuterium labeled stable isotopes. Supernatant were transferred into microtitre plates and 10µL injected onto a C8-column and directly into the AP I5000. AA and COX derived AA metabolites were analyzed with Analyst 1.5 using Quantitation Wizard (ABSCIEX). A background correction was made by subtracting the averaged amount of the blank spots analyzed in the same batch.
